# Geographical Origin Traceability of Tea (*Camellia sinensis*): A Comprehensive Review of Analytical Techniques, Chemometric Approaches, and Future Perspectives

**DOI:** 10.3390/foods15111936

**Published:** 2026-05-30

**Authors:** Hanbin Chen, Hang Wei, Hongyan Zhou, Ziyang Wu, Jie Pang, Ling Fang, Mengzhu Shi, Jianwei Fu

**Affiliations:** 1 Institute of Quality Standards and Testing Technology for Agro-Products, Fujian Key Laboratory of Agro-Products Quality and Safety, Fujian Academy of Agricultural Sciences, Fuzhou 350003, China; 2 Engineering Research Centre of Fujian-Taiwan Special Marine Food Processing and Nutrition (Ministry of Education), College of Food Science, Fujian Agriculture and Forestry University, Fuzhou 350002, China; 3 College of Modern Agricultural Technology, Fujian Vocational College of Agriculture, Fuzhou 350002, China; 4 The Institute of Crop Sciences, Fujian Academy of Agricultural Sciences, Fuzhou 350003, China

**Keywords:** tea, geographical origin traceability, chemometrics, machine learning, data fusion

## Abstract

The geographical origin fraud of tea is a serious challenge faced by the global tea market. This review systematically sorts out the full chain technical system from analysis and detection to data analysis in the field of tea origin traceability, reviews the traceability mechanism and application boundaries of four core technologies including stable isotopes, mineral element fingerprints, spectroscopy and mass spectrometry metabolomics, and emerging sensors, reveals the differential masking effect of the processing techniques of the six major tea types on chemical fingerprints, and systematically analyzes the methodological evolution of chemometrics and machine learning in origin discrimination. This article provides a systematic reference for understanding the overall pattern of the tea origin traceability technology system and the selection of differentiated traceability strategies for different tea types.

## 1. Introduction

Tea, as the non-alcoholic beverage with the second-highest global consumption after water, has seen consumers’ attention to its origin information evolve from country labels to a deep pursuit of “terroir”, the unique quality shaped by the combined factors of climate, soil, altitude and craftsmanship within a specific geographical unit [1]. Whether it is Chinese consumers’ obsession with the “core production area” of Longjing or the international market’s pursuit of geographical indication products like Darjeeling, Ceylon and Uji, all these confirm that “place of origin” has been internalized as a core dimension in the evaluation of tea quality [2,3,4]. The authenticity of the origin is directly related to the economic value of tea [5]. Take West Lake Longjing as an example: the first-picked tea from the core production area can be sold for several thousand dollars per kilogram, while the “Wuniuzao” from adjacent areas with a similar appearance is priced at only one-tenth or even less of the former. This huge price gradient created by geographical scarcity has given rise to a series of deviant behaviors centered on origin fraud; unscrupulous traders mix, blend or directly replace labels with non-origin tea to sell low-cost bulk tea as high-value geographical indication products [6]. This behavior not only infringes upon consumers’ rights and interests but also causes irreversible damage to the reputation of geographical indication brands and disrupts the global tea market order [7].

To curb the problem of origin counterfeiting, major tea-producing and consuming countries have successively established legal systems for geographical indication protection [8,9]. However, the “forward traceability” relying solely on paper documents, electronic tags or QR codes on packaging is highly vulnerable to tampering during circulation and is difficult to objectively verify in the terminal market [10,11]. Therefore, the development of “reverse traceability” technology based on the intrinsic chemical fingerprints of tea—tracing its geographical origin by detecting stable isotopes, mineral elements, characteristic metabolites and other objective information that cannot be subjectively manipulated—has become a key issue to be urgently addressed in the intersection of food science and analytical chemistry [12]. The core logic of reverse traceability technology lies in establishing a robust mapping between detection data and geographical origin labels. However, the chemical composition of tea is influenced by multiple factors such as variety, origin and processing, making traditional univariate statistical methods inadequate for accurately predicting the origin [13]. Machine learning can autonomously learn and extract implicit discriminative features from high-dimensional complex data, especially ensemble learning and deep learning architectures, which show significant advantages in dealing with issues such as variable collinearity and overfitting in small samples [14,15,16].

**Figure 1:**
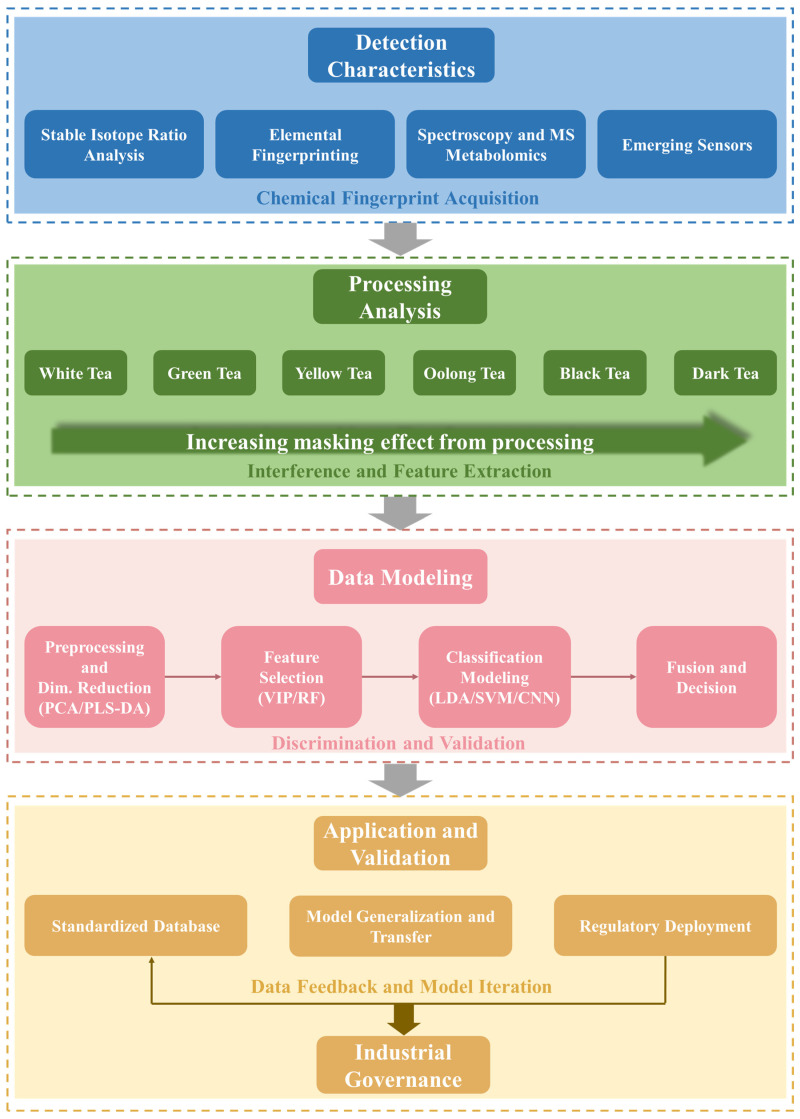
Overall review framework: detection, processing, data modeling, and application in tea origin traceability.

The research on the geographical origin traceability of tea has a distinct interdisciplinary imprint. From the agronomy and soil geochemistry at the sample collection end, to the analytical chemistry and instrument science at the detection and analysis end, to the chemometrics and computer science at the data analysis end, and finally to the food supervision and regulatory standards at the application end, the isolated research of any single link is difficult to support a complete and reliable origin identification system [17,18,19,20]. In view of this, this review, based on the achievements of multidisciplinary cross-research, strives to construct a systematic review framework that runs through the entire process of ‘detection and characterization–processing analysis–data modeling–application verification’. Figure 1 presents the overall framework of this review, which follows a logical progression from analytical detection and characterization, through processing interference analysis and data modeling, to the final application in geographical authentication. Unlike previous reviews that focus mainly on individual analytical platforms or tea quality evaluation, this review emphasizes the interaction between analytical fingerprints, processing-induced signal masking, and model validation. Particular attention is given to how tea type, fermentation intensity, aging, cultivar, harvest season, and inter-annual variability affect the robustness of geographical-origin models.

## 2. Core Technical Principle of Traceability for Tea Origin Sourcing

**Figure 2:**
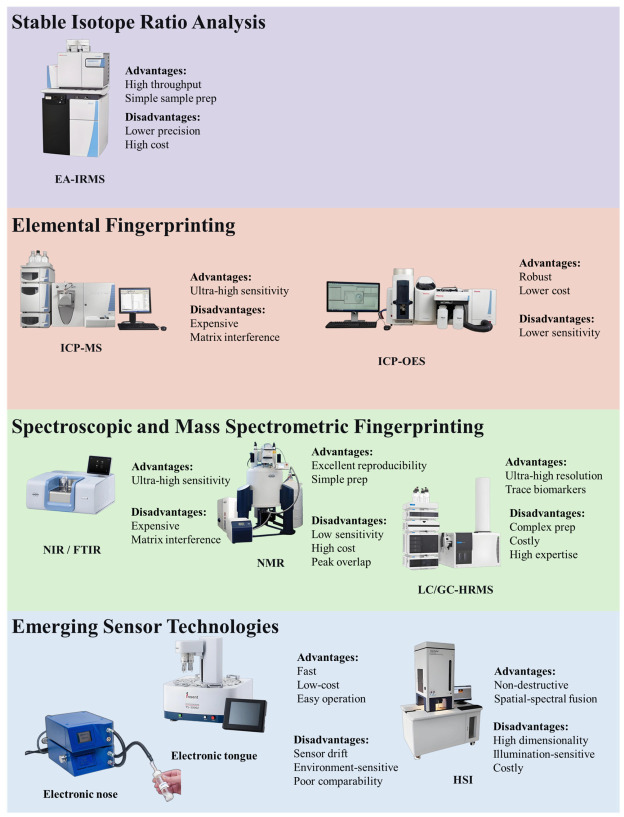
Instrumentation, advantages, and disadvantages of core analytical techniques for tea origin traceability.

The technical system for tracing the origin of tea has developed over several decades, evolving from early sensory experience discrimination to multi-dimensional chemical fingerprint analysis centered on instrumental analysis [21,22,23,24]. Starting from the physical and chemical nature of traceability information, the existing methods can be roughly classified into four categories: the first is stable isotope ratio analysis, which traces the geographical background through the regional isotopic signals of environmental water, soil and atmosphere; the second is mineral element fingerprinting, which builds a specific combination pattern of the origin by taking advantage of the selectivity of element migration in the soil–plant system; the third is spectral and mass spectrometry fingerprinting technology, which rapidly captures and finely analyzes the overall metabolic profile of tea by virtue of high information abundance; the fourth is emerging sensing technologies such as electronic nose/tongue and hyperspectral imaging, which provide portable and high-throughput supplementary solutions from the dimensions of bionic perception and spatial visualization [25,26,27,28,29]. A comparative overview of these four technology categories, including their typical instrumentation, advantages, and limitations, is provided in Figure 2. Table 1 presents a summary of key analytical techniques for tea origin traceability, arranged according to technology category. These four types of technologies have their own strengths and weaknesses, and in practical applications, they often complement each other rather than replace each other. This section will successively review their principal basis, key influencing factors and typical application effectiveness, with the aim of laying a methodological foundation for the subsequent discussion on tea-specific analysis and data interpretation strategies.

### 2.1. Stable Isotope Ratio Analysis

Stable isotope ratio analysis is one of the most widely used techniques in food geographical traceability. Its traceability logic is rooted in the spatial heterogeneity of isotope fractionation; the stable isotope composition of environmental water, carbon dioxide and nutrients absorbed by organisms shows regional distribution due to differences in latitude, altitude, distance from the coastline, geological background and climatic conditions, thereby encoding the origin information in the chemical fingerprints of biological tissues [54].

The current mainstream platform for stable isotope analysis of tea is the elemental analyzer–isotope ratio mass spectrometry (EA-IRMS) system, which mainly relies on the collaborative interpretation of four types of light stable isotopes: carbon (δ^13^C), nitrogen (δ^15^N), hydrogen (δ^2^H), and oxygen (δ^18^O) [55]. As a C_3_ plant, tea trees have δ^13^C values ranging from −32‰ to −25‰, which are regulated by environmental factors such as altitude, water, and light. Processing-induced thermal degradation may cause kinetic fractionation of carbon isotopes [56]. The δ^15^N value reflects the source of soil nitrogen and microbial transformation. The application of organic fertilizers, due to ammonia volatilization, enriches ^15^N, resulting in significantly higher δ^15^N values in tea from organic fertilizer regions compared to those from chemical fertilizer regions [57]. Liu et al.’s study on West Lake Longjing tea confirmed that carbon and nitrogen isotopes can effectively capture the origin signal [31]. δ^2^H and δ^18^O, as hydrogeographic indicators, follow the global precipitation isotope gradient shaped by latitude, altitude, continentality, and rainfall effects, and they change in concert along the global meteoric water line (δ^2^H = 8 × δ^18^O + 10) [58]. Recent studies have further found that the δ^2^H value of tea lignin methoxyl groups systematically decreases with increasing altitude, which can serve as a high-resolution altitude traceability proxy [59]. Strontium isotopes (^87^Sr/^86^Sr) are controlled by the lithology and age of geological bedrock and do not undergo biological fractionation, providing a geological fingerprint independent of climatic factors. However, their application in tea traceability is still limited at present, mainly due to the high analysis cost and complex pretreatment [60].

The stable isotope fingerprint of tea is not a static label but a dynamic signal influenced by three factors: the environment of the production area, processing techniques, and plant physiological conditions. The contribution of the production area is usually dominant. Li et al.’s research on Pu’er tea confirmed that the effect of the production area has the highest explanatory power for the variation of δ^13^C, δ^15^N, δ^2^H, and δ^18^O [34]. The interference effect of processing techniques varies depending on the intensity of tea processing and the object of measurement. Pilgrim et al. found that the impact of harvest and processing on isotope fractionation is limited, and the characteristics of the original production area can still be effectively retained after processing [30]. In addition, leaf maturity and harvest time also constitute significant covariates. Liu et al. discovered that the hydrogen and oxygen isotope ratios of West Lake Longjing tea change significantly with leaf age, and Xia et al. further revealed that the isotope values of early spring tea show a seasonal enrichment pattern, while those of late spring tea show a depletion pattern [31,33]. These studies collectively indicate that only by integrating the dominant role of the production area, the interference of processing techniques, and the variation in physiological timing into a unified framework can a robust isotope traceability model be constructed across different scenarios.

### 2.2. Mineral Element Fingerprint Spectrum

Mineral element fingerprinting is one of the most widely used techniques in the traceability of tea origin. Its core logic lies in the fact that the abundance and combination characteristics of elements in the soil parent material of different production areas are absorbed by the tea tree root system and eventually form an element distribution pattern with origin specificity in the tea tissue [61,62]. As a perennial woody plant, the tea tree’s root system is fixed in a specific geological unit for a long time, encoding the local geochemical signal in the elemental composition of the leaves. The origin indication efficacy of mineral element fingerprints depends on two premises: first, the elemental composition of the soil in different production areas varies measurably due to differences in parent rock type, weathering degree, soil formation process, and artificial fertilization; second, the element migration from soil to tea has a traceable regularity, allowing the soil characteristics to be “transcribed” into the tea tissue, forming an origin-specific chemical barcode [37].

Mineral element fingerprints typically cover major elements (Ca, Mg, K, P), trace elements (Zn, Cu, Mn, Fe, etc.) and rare earth elements (La, Ce, Pr, Nd, etc.), with the number of detected elements ranging from a dozen to over sixty. Inductively coupled plasma mass spectrometry (ICP-MS), due to its high sensitivity and ability to simultaneously measure multiple elements, has become the standard for this technology; inductively coupled plasma optical emission spectrometry (ICP-OES) is often used for major element analysis and complements ICP-MS to achieve full elemental coverage. The core advantage of mineral element fingerprints lies in their processing and storage stability; the elemental composition is less affected by processing, making it particularly suitable for model construction across different tea types and years.

In mineral element fingerprinting, alkali metals and alkaline earth metals (Rb, Cs, Sr, Ba, Mg, Ca) are the most frequently identified traceability variables due to their close relationship with the evolution of geological bodies. Research in the tea-growing areas of northeastern India has confirmed that Rb, Cs, Sr, Ba, and Mg show significant differences among different production areas [63]. Transition metals (Cr, Mn, Co, Ni, Cu, Zn, Mo) are influenced by both geological background and human activities, and can comprehensively reflect the natural and human geographical characteristics of the production areas. Kanrar et al.’s ICP-MS analysis of 321 samples indicated that transition metals such as Mn, Zn, and Cu show significant differences among production areas and are core components of the element fingerprint [64]. Rare earth elements (La, Ce, Pr, Nd, Sm, Eu, Gd, etc.) occur in stable combinations in nature and have limited fractionation during soil–plant transfer, thus they can well preserve the geochemical characteristics of the source area. Dongting Biluochun tea achieved a recognition rate of 98.2% and a prediction ability of 96.4% based on 37 elements combined with the LDA model [36]. Heavy metal elements (Pb, Cd) are affected by both parent material and atmospheric deposition, and the differences among production areas with different degrees of industrialization can serve as effective origin signals. Liu et al. identified ^86^Sr and ^112^Cd as markers for tea origin classification, and Zhao et al. confirmed that Na, Mg, Ca, Ni, Rb, Sr, and Pb are efficient traceability descriptors [37,65].

The contribution of geographical origin to the elemental composition of tea usually outweighs other factors such as variety and cultivation measures. Girolametti et al.’s analysis of tea garden samples from multiple European countries confirmed significant differences in elemental profiles among different production areas; Zhu et al. found that there were 35 and 31 elements with significant differences among the three major production areas of ripe Pu’er tea, and Mo, Nd, Ce, Sr, Ba, V, and Tm were identified as the most discriminative elements [39,66]. The effectiveness of mineral element fingerprints highly depends on the migration patterns of elements from soil to tea leaves. Zhang et al.’s analysis of 87 tea samples and corresponding soils from three production areas confirmed that 17 elements showed significant correlations between soil and tea leaves [67]. The interference effects of variety and processing techniques vary with the intensity of tea processing: Zhu et al. found that piling fermentation in Pu’er tea led to significant enrichment of 36 elements, with enrichment factors of Ti, Cs, Nb, etc., reaching 1.55–2.02 times; while Fernández-Cáceres et al.’s study on green tea and black tea indicated that the elemental fingerprints of lightly fermented tea types were relatively less affected by processing [35,39]. Therefore, in cross-tea type traceability, element combinations that are less sensitive to processing and variety should be prioritized, or correction factors should be introduced to enhance the robustness of the model.

Although the mineral element fingerprint is robust to processing techniques, the impact of inter-annual climate fluctuations on model performance has not been systematically evaluated. It should be noted that the high classification accuracy of mineral element fingerprints in the current literature is usually established under the premise of sufficient sample size, significant geological background differences among production areas, and strict validation strategies. When the geological homogeneity of production areas is high or the sample size is limited, the model performance may decline significantly. At this time, stable isotopes or metabolomics should be combined for complementarity.

### 2.3. Spectral and Mass Spectrometry Fingerprinting Techniques

Spectral and mass spectrometry fingerprinting techniques are the most dynamic technology clusters in the field of tea origin traceability. By capturing the overall response signals of samples under energy excitation, high-dimensional “spectral fingerprints” or “metabolite fingerprints” are constructed, and the origin discrimination information is extracted with the aid of chemometrics and machine learning [68,69]. The core advantage lies in the extremely high information abundance; hundreds to tens of thousands of variables can be obtained in a single measurement, covering a wide range of chemical spectra from primary metabolites to secondary metabolites. This is particularly suitable for complex origin differences that are difficult to capture with traditional targeted methods [70,71].

Near-infrared spectroscopy (NIR, 780–2500 nm) and mid-infrared spectroscopy (MIR, 2500–25,000 nm) can rapidly capture the overall chemical information of tea by detecting the overtone and combination absorption of molecular vibrations [72]. The NIR signal mainly originates from the vibrations of hydrogen-containing groups (O–H, C–H, N–H), reflecting the comprehensive characteristics of components such as moisture, tea polyphenols, amino acids, and sugars; FTIR is more sensitive to polar functional groups in the mid-infrared band [73,74,75]. Li et al., based on the FTIR and NIR data of 360 black tea samples from 9 global production areas, achieved 100% cross-validation and external validation accuracy with SVM and KNN models on FTIR [46]. The combination of multispectral fusion and deep learning is an important development direction; Chen et al. proposed an ECA-ResNet model that fuses Raman and NIR features, achieving a traceability accuracy of 95.05% for Pu’er tea [47]. NIR/FTIR technology is rapid, non-destructive, and portable, but it has problems such as moisture sensitivity, weak interpretability, and limited cross-year migration. Hong et al.’s study on 1447 cross-year samples of FT-NIR confirmed the need to introduce model update strategies to address feature drift [76]. Currently, most studies focus on specific production areas, and the construction of a shared spectral database covering all tea types and across production areas remains a key bottleneck restricting the practical deployment of this technology.

In addition to benchtop instruments, portable and handheld spectrometers have gained increasing attention for rapid, on-site, and low-cost geographical origin authentication of tea. These portable systems are typically based on miniaturized near-infrared, Raman, or smartphone-coupled platforms, offering clear advantages in field deployability and real-time screening along the supply chain, albeit with generally lower spectral resolution and signal-to-noise ratio compared with laboratory-grade instruments. Multiple studies have demonstrated the feasibility of portable NIR technology for tea origin classification. For instance, Ferreira et al. showed that a portable NIR (PNIR) instrument achieved F1 scores exceeding 99% for black tea samples from Brazil, the United States, and India, performing comparably to benchtop NIR systems [77]. Similarly, Jin et al. reported that a field-portable spectroradiometer coupled with a support vector machine model achieved an average accuracy of 98.9% in distinguishing Anji Baicha samples from core versus other production regions [78]. Thus, portable spectrometers offer a viable, cost-effective alternative for tea origin authentication, bridging the gap between laboratory precision and field screening needs.

Nuclear magnetic resonance spectroscopy (^1^H NMR) plays a central role in the origin tracing driven by metabolomics. Its principle is based on the resonance signals of ^1^H and other spin nuclei in a strong magnetic field [79]. Chemical shift, coupling constant, and peak area provide information on molecular structure, functional group connection, and relative content, respectively. ^1^H NMR has a universal response to all hydrogen-containing compounds, and the signal intensity is linearly related to concentration, allowing for accurate quantification without the need for standard calibration. From a foodomics perspective, NMR spectroscopy is particularly advantageous for authenticity and quality assessment, as it yields highly reproducible, information-rich fingerprints with minimal sample preparation and exhibits excellent compatibility with chemometric and machine-learning workflows. In this context, Ciampa et al. emphasized that NMR-based metabolomics enables a more holistic and sustainable evaluation of food quality by integrating compositional data with multivariate analysis, thereby improving the interpretation of food composition, authenticity, and overall quality [80]. In the ^1^H NMR analysis of 78 Longjing tea samples by Hou et al., the random forest model achieved a classification accuracy of 92.2% for PDO and non-PDO regions, outperforming the 85.6% accuracy of linear discriminant analysis, and identified 15 origin markers such as kaempferol glycosides and glutamine [45]. The advantages of ^1^H NMR lie in its high reproducibility and quantitative accuracy, but it has relatively low sensitivity, high instrument cost, and severe peak overlap, which limit the precise assignment of metabolites [81]. In the future, two-dimensional NMR techniques should be expanded, mass spectrometry data integrated, and standardized processes unified to promote their application in regulatory contexts.

High-resolution mass spectrometry (HRMS) combined with chromatographic separation techniques represents the standard technical route for the metabolomic traceability of tea origins [82]. It has a significantly higher sensitivity than NMR and can detect low-abundance secondary metabolites, but it involves complex sample pretreatment and a long analysis time. In the field of black tea traceability, Li et al. analyzed 302 samples from 9 global production areas using LC-QToF and identified 229 and 145 origin biomarkers, achieving a 100% classification accuracy rate in both internal 7-fold cross-validation and external validation [83]. In the narrow geographical scale traceability of green tea, Zhang et al. used UHPLC-QTOF-MS to conduct a fine discrimination between the first and second grade production areas of West Lake Longjing, identifying 20 differential metabolites, with a prediction accuracy of 99% as determined by Monte Carlo simulation [43]. In the field of dark tea, Su et al. conducted a non-targeted metabolomic analysis of 47 dark tea samples using UHPLC-Q-Exactive Orbitrap MS, screening out 12 key origin marker metabolites dominated by altitude, achieving a 100% accuracy rate in both the training and validation sets [84]. These examples collectively demonstrate the power of HRMS metabolomics for geographical authentication. Consistent with this view, García-Pérez et al. emphasize that metabolomics and chemometrics function as complementary tools: NMR- and MS-based metabolomics generate comprehensive chemical fingerprints, whereas multivariate and machine-learning methods are essential to extract authenticity markers, disentangle interacting factors, and support robust geographical-origin classification [85]. It is worth noting that the high accuracy rates reported in narrow geographical scale traceability studies using HRMS metabolomics are often established under conditions of small sample sizes and without independent cross-year validation. The high-dimensional and small sample characteristics of non-targeted metabolomics pose a significant risk of overfitting. Additionally, HRMS metabolomics currently faces challenges such as the reliance on database completeness for metabolite identification, insufficient comparability of cross-platform data, and high analysis costs. In the future, efforts should be made to promote the formulation of standardized data collection processes, the construction of public metabolite databases, and the development of automated data analysis pipelines.

### 2.4. Other Emerging Sensing Technologies

In addition to mainstream techniques such as stable isotope, mineral element fingerprinting and spectral/mass spectrometry metabolomics, emerging technologies based on bionic sensing and spectral imaging principles have also demonstrated application potential in the origin traceability of tea.

Electronic nose (E-nose) and electronic tongue (E-tongue) are bionic sensing platforms that simulate the olfactory and gustatory systems of mammals [86]. The electronic nose forms an “aroma fingerprint” by generating cross-reactions of volatile compounds in tea through a gas sensor array, while the electronic tongue builds a “taste fingerprint” by measuring the electrochemical signals of tea soup through an electrode array [87]. Both technologies offer rapid analysis, require no complex pretreatment, and have low equipment costs, making them particularly suitable for high-throughput screening at the front end of the supply chain. Jin et al. used an electronic nose combined with HS-SPME-GC-MS to analyze Tongcheng Xiaohua tea, identifying 66 volatile metabolites and screening out 7 regional differentiation compounds. α-pinene and β-cyclocitral were identified as new regional markers [52]. Kanaga Raj et al. developed an impedance-type multi-sensor electronic tongue based on nanostructured materials and combined PLS-DA and PLSR to distinguish black tea from different origins [51]. The limitations of electronic nose/tongue technology include sensor drift, environmental sensitivity, and poor comparability across devices. In the future, it is necessary to develop stable sensing materials, establish cross-device calibration algorithms, and promote the construction of a multi-modal fusion traceability system.

Hyperspectral Imaging (HSI) integrates spectral analysis and digital image processing to generate a three-dimensional data cube containing two-dimensional spatial and one-dimensional spectral information through line-by-line scanning, achieving “image-spectrum integration”. It can simultaneously capture the spatial distribution of the appearance texture and internal chemical components of tea leaves, offering a visualization advantage in origin traceability [88]. Guo et al. combined near-infrared and hyperspectral data for the origin traceability of sun-dried green tea, achieving 100% classification accuracy with both SVM and RF models [53]. Hong and He identified the origin of single Longjing tea leaves in the 380–1030 nm and 874–1734 nm ranges, with accuracy rates over 84% for both the calibration and prediction sets, and for the first time generated a geographical origin prediction distribution map [49]. Liu et al. used NIR-HSI combined with PCA-SVM to simultaneously discriminate the geographical origin and processing month of green tea from three production areas in Chongqing, achieving prediction accuracies of 97.5% and 95%, respectively [50]. The advantages of HSI lie in its non-destructiveness, high information density of “image-spectrum integration”, and visualization of origin information. However, it faces challenges such as high data dimensionality, sensitivity to lighting, and high cost. Future efforts should focus on the development of portable devices, the integration of deep learning, and the construction of standardized image databases.

Electronic nose and tongue, as well as hyperspectral imaging, complement each other in tracing the origin of tea from the dimensions of sensory profile and spatial distribution. Both have the advantages of being rapid, non-destructive, and high-throughput screening, and combined with machine learning, they can achieve a classification accuracy of over 90%. Recent sensor-oriented studies further indicate that portable, non-destructive sensing platforms are becoming increasingly relevant for food authentication. In this context, Picone highlighted that integrating metabolomics and machine learning can improve the identification and interpretation of complex chemical signatures in complex matrices–a perspective directly relevant to tea origin traceability, where emerging sensors generate high-dimensional fingerprints that require robust preprocessing, classification, and validation [89]. The common bottleneck lies in insufficient standardization; the attenuation of sensor sensitivity, lighting sensitivity, and poor comparability across devices has restricted their practical deployment. In the future, it is necessary to formulate standardized collection and correction procedures, develop low-cost portable devices, and promote the fusion of multi-modal data to achieve the large-scale application of emerging sensing technologies in the origin tracing of tea.

## 3. Research Status and Specific Challenges of Origin Traceability for Different Tea Categories

**Figure 3:**
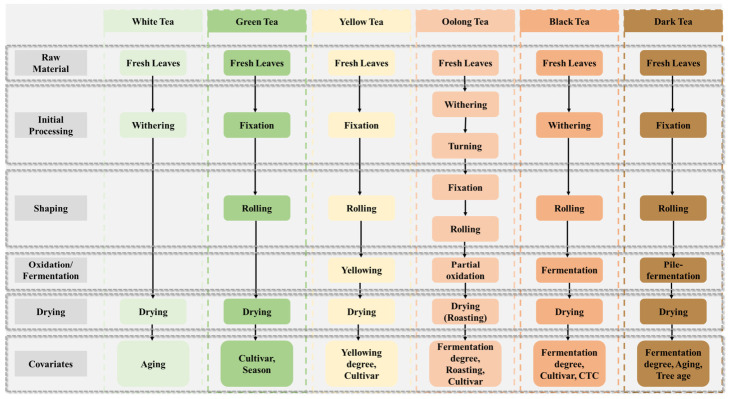
Processing workflows and key covariates affecting origin traceability for the six major tea categories.

The origin traceability of tea is not merely an analytical chemistry issue. The essential differences in the processing techniques of the six major tea types determine that the applicability boundaries of the same traceability technology may vary significantly among different tea types. A comparative illustration of the processing workflows and key confounding factors for green, white, yellow, oolong, black, and dark teas is provided in Figure 3. The high-temperature kill-green process in green tea deactivates the polyphenol oxidase activity, preserving the original chemical fingerprint of the fresh leaves to the greatest extent. White tea undergoes only withering and drying, with the least processing intervention, and theoretically has the best traceability. Yellow tea adds a “yellowing” process on the basis of green tea, and the impact of the micro-fermentation process on the chemical profile remains to be systematically evaluated. Oolong tea’s withering and roasting processes result in a fermentation degree ranging from 10% to 70%, making the complexity of the chemical fingerprint’s interference from processing the most among the six major tea types. The full fermentation process in black tea converts a large amount of catechins into theaflavins and thearubigins, deeply reshaping the primary metabolite profile. The post-fermentation of dark tea, driven by microbial communities, triggers biochemical transformations that can last for months or even years, with the origin signal highly intertwined with factors such as the degree of fermentation, aging years, and tree age [90,91,92,93,94,95,96]. For this reason, discussing traceability without considering the specificity of tea types is equivalent to ignoring the masking effect of processing techniques on the chemical fingerprint. The degree of processing should therefore be treated as a primary covariate in tea-origin traceability: lightly processed teas are more suitable for metabolite-based origin discrimination, whereas heavily fermented or post-fermented teas require more robust markers such as mineral elements, stable isotopes, or multi-source data fusion. Therefore, this section will systematically review the origin traceability research of different tea types based on processing characteristics, aiming to provide a differentiated reference framework for the selection of traceability strategies for different tea types.

### 3.1. Green Tea

Green tea is the most widely consumed type of tea globally. The core of its processing lies in the high-temperature blanching to inactivate the polyphenol oxidase activity and preserve the natural chemical components of the fresh leaves. The processing steps of green tea are simple and have a low degree of interference with the chemical fingerprint, making it the tea type with the richest data accumulation in origin traceability research.

The first and second grade production areas of West Lake Longjing are geographically adjacent and have similar climates, providing a strict validation scenario for micro-scale traceability. Deng et al. combined δ^13^C and mineral elements with a random forest model to distinguish West Lake Longjing from other production areas, achieving an accuracy rate of 97.6% [97]. Anji White Tea represents the most systematic application of mineral element fingerprints in green tea. Zhu et al. conducted ICP-MS/OES analysis on 365 samples for three consecutive years, and the SVM model achieved a prediction accuracy rate of 92.7% on the independent test set. OPLS-DA identified Mo, Cu, and Rb as key discriminant elements, and confirmed that Rb, Mn, Pb, Mg, and K have stable migration correlations between soil and tea (R^2^ > 0.5) [40]. The design of continuous sampling for three years in this study effectively controlled the interference of inter-annual climate fluctuations on the model’s robustness, while most current green tea traceability studies are still limited to a single year, and the generalization performance across years remains to be verified. The origin traceability of Japanese green tea mainly focuses on distinguishing domestic from imported tea and inter-county discrimination within the country. Kohata et al. achieved effective classification between countries and counties based on the analysis of ten element contents [98].

The core of green tea processing is high-temperature blanching to inactivate polyphenol oxidase activity [99]. Its interference with the chemical fingerprint mainly lies in the changes in the content of heat-sensitive metabolites due to thermal degradation, the rearrangement of volatile aromas caused by rolling and drying, and the impact of different blanching methods on the metabolic profile. However, in general, the masking effect of green tea processing on the chemical fingerprint is much weaker than that of fermented tea types, and the main regional discrimination markers are usually effectively retained after processing.

### 3.2. White and Yellow Tea

The processing techniques of white tea and yellow tea are mild, and the degree of fermentation is relatively low. White tea only undergoes withering and drying, with the least processing intervention; yellow tea, based on the processing of green tea, adds a “yellowing” procedure and is a slightly fermented tea. The chemical fingerprints of these two types of tea are less affected by processing, and theoretically, they have superior traceability of origin. However, the actual research accumulation is far behind that of green tea, black tea and oolong tea.

White tea mainly originated in Fujian Province, China. In recent years, large-scale production has also started in Yunnan, Guizhou, and Sichuan. Near-infrared spectroscopy is one of the most mature technologies used in the origin traceability of white tea. Zhang et al. constructed three KNN discrimination models for 579 samples of Bai Mudan tea, including provincial discrimination, county-level discrimination, and authenticity identification of Fuding tea, with accuracy rates of 88.97%, 93.88%, and 97.96%, respectively [100]. This study had a sufficient sample size and a reasonable validation stratification, but it did not involve cross-year validation; the accumulation of flavonoids and alkaloids during the aging process of white tea may affect spectral characteristics, and the applicability of the model to aged white tea remains to be verified. The application of mineral element fingerprints in the origin traceability of white tea began earlier. Ye et al. analyzed 26 elements in 64 samples from the three major production areas of Fuding, Zhenghe, and Jianyang, and the recognition accuracy rates of LDA, SVM, and KNN models reached 98.44%, 95.31%, and 100%, respectively, but the sample size was small and there was no independent external validation, and the robustness of the conclusion’s extrapolation needs to be verified [101]. In terms of large-scale traceability across regions, Zhao et al. analyzed the chemical composition and sensory quality of Bai Mudan tea from three production areas in Fujian Fuding, Henan Xinyang, and Yunnan, and found significant differences in chemical composition between the emerging production areas and the traditional Fujian production areas that could be modeled. They identified 12 characteristic compounds, including gallic acid, theaflavin, and L-glutamic acid, as key contributing factors [102].

White tea undergoes only withering and drying, with the least processing intervention among the six major tea types. Its masking effect on chemical fingerprints is the weakest, allowing the effective retention of geographical origin markers during processing. However, long-term aging can cause significant chemical changes; EPSFs and other flavonoid alkaloids accumulate with aging and have been identified as specific markers of storage duration [103]. Additionally, the moderate oxidation by polyphenol oxidase during withering results in significantly higher flavonol/flavonoid glycoside content in white tea compared to other tea types, while catechins and anthocyanins are significantly lower than in green tea and close to those in black tea [104]. Therefore, when using catechin metabolites for geographical origin discrimination, it is necessary to be cautious of the interference introduced by batch differences in withering degree.

Yellow tea has a small production volume and is mainly produced in concentrated areas such as Hunan, Sichuan, and Anhui, with distinct regional specificity. Research on the origin tracing of yellow tea is still in its infancy, mainly due to the small scale of the industry and insufficient academic attention. The characteristic process of yellow tea, “yellowing”, triggers non-enzymatic oxidation of polyphenols, hydrolysis of ester catechins, and degradation of chlorophyll through wet heat after the processes of pan-frying and rolling. Metabolomics studies have confirmed that this process leads to significant changes in the content of non-volatile metabolites such as catechins, amino acids, and phenolic acids [105,106]. The degree of modification of the metabolic profile by yellowing is stronger than that of withering in white tea and pan-frying in green tea, but much less than full fermentation in black tea and post-fermentation in dark tea. Therefore, the origin signals at the metabolite level may be partially masked by processing signals, and the variety differences further complicate the interwoven variables. In contrast, mineral elements and stable isotope fingerprints are not sensitive to wet heat oxidation, suggesting that they can be effectively retained before and after yellowing and should be the preferred technical route for origin tracing of yellow tea [35].

### 3.3. Oolong Tea

The processing technology of oolong tea is the most complex, with the degree of fermentation ranging from light (10–20%) to medium (30–50%) and then to heavy (60–70%), covering a semi-continuous spectrum from green tea to black tea [107]. Its quality is highly dependent on the “terroir” of specific geographical units, with the price of core and non-core production areas differing by several times or even tens of times [108]. At the same time, complex processes such as “doing the green” and “roasting” have a significantly stronger masking effect on chemical fingerprints than green tea and white tea, presenting a challenge of high demand and high technical difficulty for the traceability of oolong tea.

Wuyi rock tea is the core category of oolong tea with the strictest geographical indication protection, produced in Wuyi Mountain, Fujian Province. The quality grade classification of “zhengyan”, “banyan” and “zhou tea” of Wuyi rock tea has formed a very narrow geographical scale discrimination requirement. Peng et al. conducted GC-TOF-MS analysis on 333 samples of Ruiyan, and the MLP model based on 176 volatile features achieved an average accuracy rate of 92.7%, with the independent test set exceeding 90% [44]. However, the generalization ability of the model across varieties and years needs to be verified. Lou et al. combined stable isotopes such as δ^2^H and δ^18^O with mineral elements, and the SVM model achieved an accuracy rate of 97.73% in discriminating the origin of Wuyi rock tea, with δ^2^H, δ^18^O, Cs, Cu, Ca and Rb identified as core contributing variables [109]. Jin et al. found that stable isotopes had a limited effect in distinguishing Tieguanyin from Da Hong Pao, while the combination of GC-IMS and k-NN model achieved an accuracy rate of 86.7% in discriminating sub-regions of Da Hong Pao, and the overall classification accuracy rates for Tieguanyin and Da Hong Pao were 95.2% and 97.8%, respectively, indicating that volatile omics has unique advantages in the discrimination of oolong tea varieties [110].

Anxi Tieguanyin is a representative category of oolong tea, originally from Anxi, Fujian Province. As the cultivation area expanded, the problem of “counterfeit origin” has given rise to a large number of traceability studies. Yan et al. were the first to verify the feasibility of rapid identification of Tieguanyin origin by combining near-infrared spectroscopy with PLS-DA [41]. Meng et al. further integrated ^1^H NMR with NIR, achieving a discrimination accuracy of 86.2–95.8% for 90 samples from three origins in Fujian, but the sample size was small, and there was no independent external validation, and the generalization performance across regions remains to be verified [42]. In a study targeting Huangguanyin, ICP-MS was used to determine 15 mineral elements and 15 rare earth elements, and the PCA-SVM model based on differential elements achieved a classification accuracy of 100% for samples from Yunxiao and Wuyishan. However, among the 31 chemical components identified by targeted metabolomics, only 14 showed origin differences, and the PCA-SVM recognition efficiency was 88.89% [111]. This comparison reveals that the mineral element fingerprint is more robust than the metabolite fingerprint in the traceability of oolong tea origin.

Taiwan is a significant global producer of oolong tea. Wang et al. utilized HS-SPME/GC-MS to analyze the volatile components of Taiwanese Dongfangmeiren tea. The OPLS-DA model identified 37 differential metabolites for regional discrimination and pinpointed 8 key volatile components highly consistent with sensory quality, including linalool-related compounds [112]. This study demonstrated the dual application value of volatile profile analysis in the identification of oolong tea origin and quality assessment. Wu et al. employed VIS/NIR spectroscopy combined with SVM to discriminate partially fermented teas from Vietnam, mainland China, and Taiwan. The full-wavelength model achieved 100% classification accuracy for tea type, country of origin, and sub-regions within Taiwan in both calibration and prediction sets [107]. Kaushal et al. utilized an electronic nose coupled with machine learning to classify Jinxuan oolong tea from four origins: Taiwan, Vietnam, mainland China, and Indonesia. The LDA and ANN models attained an overall accuracy of 98.33% [108].

The processing of oolong tea is complex, involving withering, green-making, pan-frying, rolling, drying and multiple roasting. The masking effect on chemical fingerprints mainly occurs at three levels. Firstly, enzymatic oxidation during green-making is the core interfering factor; the fermentation degree of oolong tea ranges from 10% to 70%, and the differences in polyphenol composition under different fermentation degrees may mask the origin signal. Reyrolle et al. confirmed that the correlation between VOC fingerprints and variety and processing techniques is significantly higher than that of geographical origin [113]. Therefore, when using volatile metabolites for origin discrimination, the fermentation degree and variety variables need to be strictly controlled. Secondly, the roasting process generates a large number of volatile components through the Maillard reaction and changes the content of heat-sensitive metabolites, interfering with the metabolite fingerprint, while mineral elements and stable isotopes are basically insensitive to this. Thirdly, the variety covariate is particularly prominent in the traceability of oolong tea. Existing studies have revealed that the influence of variety factors on the metabolic profile may overwhelm the origin signal [44,114]. Future oolong-tea traceability studies should therefore adopt stratified sampling designs that explicitly record cultivar, fermentation degree, roasting intensity, harvest season, and storage status. Without such metadata, models may capture processing or cultivar differences rather than true geographical-origin signals. In the future, it is necessary to systematically evaluate the interaction effect between variety and origin or build a multi-variety large sample database to enhance generalization ability.

### 3.4. Black Tea

Black tea is the most produced tea type worldwide. The core of its processing lies in “fermentation”, where catechins, under the catalysis of polyphenol oxidase, oxidize and polymerize into characteristic products such as theaflavins and thearubigins. This full fermentation process significantly alters the chemical fingerprint more than any other tea type, with primary metabolites being largely transformed and aroma components undergoing drastic rearrangement. Therefore, the core challenge in the origin traceability of black tea lies in identifying and preserving the robust chemical signals associated with geographical origin from the deep processing.

Qimen black tea is renowned for its “Qimen aroma”, with its core production area located in Qimen County and traditional production areas including Dongzhi and Guichi. Peng et al., based on EA-IRMS analysis of δ^13^C and δ^15^N, found that variety and leaf maturity affected δ^15^N, while processing techniques had no significant impact. The k-NN model achieved a cross-validation accuracy rate of 91.6% for the core and adjacent production areas [32]. Ren et al. used ICP-MS to determine 27 elements in 104 samples, and both LDA and SVM achieved a 100% classification accuracy rate for the three production areas. Ten elements, including Rb, Ba, and Sr, were confirmed as core contributing factors [38]. Peng et al., based on UHPLC-Q/TOF-MS non-targeted metabolomics to discriminate five township-level production areas within Qimen County, identified 39 differential metabolites, and the FNN model achieved a 100% recognition accuracy rate [115]. Yun et al., based on static headspace GC-MS combined with k-NN/RF models, achieved a discrimination rate of 95–100% for the test set of black teas from China, India, and Sri Lanka [116]. The above studies collectively constructed a hierarchical traceability system for Qimen black tea, providing a systematic reference for the technical path selection of other black tea production areas.

The full fermentation of black tea profoundly transforms its chemical fingerprint, with masking effects manifesting in four aspects. Firstly, the oxidation and polymerization of catechins into theaflavins, thearubigins, etc., fundamentally alter the polyphenol profile; the content of flavonols/flavonoid glycosides in black tea significantly increases, while catechins and anthocyanins significantly decrease, leading to a decline in the signal-to-noise ratio of the origin discrimination model based on catechins. Secondly, the CTC process more thoroughly destroys the cells, changing the release pattern of volatile aromas and interfering with the aroma fingerprint discrimination. Thirdly, Cui et al. confirmed through ^1^H NMR studies that the contribution of variety to the metabolic fingerprint of black tea is greater than that of processing techniques, and the signals of origin and variety are highly intertwined [117]. Fourthly, mineral elements and stable isotopes are insensitive to fermentation and CTC processing, and the origin characteristics can be effectively retained after processing [30,32]. Therefore, for the traceability of black tea, mineral elements and stable isotopes should be prioritized, and metabolomics should be used as a precise confirmation method under the condition of consistent varieties. This principle may be extended to other strongly processed teas: when enzymatic oxidation, microbial post-fermentation, or thermal treatment profoundly reshapes the metabolome, origin models should prioritize markers that are less sensitive to processing and use metabolomics primarily for confirmatory or fine-scale discrimination.

### 3.5. Dark Tea

The defining characteristic of dark tea lies in “post-fermentation”, a complex series of microbially driven biochemical transformations occurring after fixation, rolling, and initial drying, either through artificial pile-fermentation or prolonged natural aging. Dark tea is broadly classified into Yunnan Pu-erh tea (including raw sheng and ripened shou varieties) and various regional dark teas such as Hunan Anhua dark tea, Hubei Qingzhuan brick tea, Sichuan Tibetan tea, and Guangxi Liupao tea. During post-fermentation, microbial metabolism and organic matter degradation profoundly alter the chemical fingerprint, resulting in a tight intertwining of origin signals with factors such as fermentation degree, aging duration, and tree age. Consequently, origin traceability for dark tea faces the dual challenges of “signal attenuation” and “covariate interference”.

Pu’er tea is the category with the most accumulated research in the origin traceability study of dark tea. The traceability demands for Pu’er tea are mainly focused on three aspects: distinguishing between different production areas, differentiating between ancient tree tea and terrace tea, and identifying the ripeness and aging years of the tea. In terms of stable isotopes, Li et al. analyzed the δ^13^C, δ^15^N, δ^2^H, and δ^18^O of whole tea and caffeine monomers. Variance decomposition confirmed that the contribution rate of the origin factor to the variation in each index was the largest. Among them, δ^13^C~caffeine~, δ^13^C~tea~, δ^15^N~tea~, and δ^2^H~caffeine~ were the key variables for origin discrimination. However, δ^2^H~tea~ and δ^13^C~caffeine~ were significantly affected by processing techniques, and the interaction effect of origin and processing was significant in all indicators [34]. This study quantitatively confirmed that the post-fermentation of Pu’er tea significantly interfered with the isotopic fingerprint. In terms of elemental fingerprinting, Zhu et al. found that the piling fermentation of Pu’er tea led to a general enrichment effect of elements [39]. Chen et al. systematically investigated the interactive effects of origin and tree age on the stable isotopes and multiple elements of Pu’er tea. They found that the Mn content was significantly affected by the interaction of origin and tree age, but 24 parameters were closely related to origin rather than tree age. Only six parameters were needed to achieve a 100% cross-validation accuracy rate [118]. This study indicated that the interference of tree age on isotopic and elemental fingerprints could be effectively controlled by screening parameter combinations with high robustness to tree age. In metabolomics, Wu et al. used ^1^H NMR and UHPLC/Q-TOF-MS to discriminate the origin of raw Pu’er tea, and sPLS-DA selected valine, threonine, chlorogenic acid, etc., as key contributing variables [119]. In volatilomics, Wang et al. used HS-SPME-GC-MS combined with machine learning to analyze raw Pu’er tea from 10 production areas in Yunnan. A random forest model based on five key volatiles, such as pentanal and heptanal, achieved a discrimination accuracy of 98.4% [120]. The above studies jointly constructed a hierarchical traceability system for Pu’er tea, providing a systematic reference for the selection of technical paths in other dark tea production areas.

The post-fermentation of dark tea has a profound and long-lasting impact on the chemical fingerprint. The masking effect is manifested in three aspects: first, the microbial metabolism and organic matter degradation during piling fermentation lead to the relative enrichment of elements; second, long-term aging continuously alters the metabolic profile; third, the age of the tea tree is an important covariate. When building the model, parameter combinations that are not sensitive to the age of the tea tree should be prioritized for selection or correction factors should be introduced.

## 4. Chemometrics and Machine Learning-Driven Data Analysis

The raw detection data cannot directly answer the question of origin. The leap from data to labels relies on the in-depth analysis of high-dimensional data by chemometrics and machine learning. The data analysis of tea origin traceability usually follows the progressive logic of “preprocessing and dimensionality reduction, feature selection, classification modeling, and fusion decision-making”. Based on this, this section will systematically review the current research status of tea origin traceability driven by chemometrics and machine learning, aiming to provide methodological guidance for the complete data analysis process of tea origin traceability from “instrument signal acquisition” to “intelligent discrimination and decision-making”.

### 4.1. Data Preprocessing and Dimensionality Reduction

In the research on the origin traceability of tea, the raw data generated by analytical instruments usually have the characteristics of high dimensionality, high noise and multiple interferences. Among these high-dimensional data, the effective information related to the origin is often masked by non-biological variations such as instrument noise, baseline drift, sample preparation differences and batch effects. Therefore, systematic preprocessing and dimensionality reduction before modeling are the key prerequisites for ensuring the robustness and interpretability of the model.

Data standardization and normalization are fundamental preprocessing steps for traceability modeling. For elemental and isotopic data, auto-scaling or Pareto scaling combined with mean-centering is routinely employed to eliminate unit and magnitude differences while preserving variance structure. For spectroscopic data, the combination of standard normal variate or multiplicative scatter correction followed by Savitzky–Golay first or second derivative and mean-centering represents the most prevalent preprocessing pipeline to correct for light scattering and baseline drift. For metabolomics data, Pareto scaling or log transformation with mean-centering is frequently adopted to handle heteroscedasticity and wide dynamic ranges. Outlier removal, most commonly guided by PCA score plots or Hotelling’s T^2^, is also a routine step to improve model robustness [100,121].

Principal Component Analysis (PCA) is the most commonly used unsupervised dimensionality reduction method in the traceability of tea origin. It projects high-dimensional data onto a few principal components through orthogonal transformation, achieving data compression and visualization. PCA serves multiple functions, including data dimensionality reduction, exploratory clustering, batch effect identification, and outlier detection. In the discrimination of green tea origin based on HPLC-DAD two-dimensional fingerprint spectra by Gu et al., the PCA score plot clearly showed the clustering trend of samples from Zhejiang and Shandong production areas, providing a feasible basis for subsequent OPLS-DA modeling [122]. Although PCA can effectively alleviate the curse of dimensionality in high-dimensional small sample problems, its unsupervised nature may cause the extracted principal components to reflect the largest variance sources rather than the differences between origins. Therefore, it is more suitable as an exploratory analysis tool rather than a final classifier.

Partial least squares discriminant analysis (PLS-DA) is the most widely used supervised dimensionality reduction method, which directly links dimensionality reduction to classification goals by maximizing the covariance between the X and Y matrices when extracting latent variables. In the traceability of Anji White Tea using an electronic tongue, the classification performance of PLS-DA was significantly better than that of PCA [48]. Permutation test is an essential means to verify whether PLS-DA is overfitting, by randomly shuffling the labels and comparing R^2^Y and Q^2^ values [123]. Orthogonal partial least squares discriminant analysis (OPLS-DA) further decomposes the variation in the X matrix into predictive components and orthogonal noise components. After filtering out the noise, it usually achieves better classification performance [122]. Variable importance in projection (VIP) is a core indicator for evaluating the contribution of variables, and variables with VIP > 1 have a significant contribution to classification [117].

Data preprocessing and dimensionality reduction are indispensable preliminary steps in the modeling chain for tea origin traceability. It is recommended to follow a progressive process of “unsupervised exploration first, then supervised modeling”: Firstly, use PCA to identify data structure and batch effects, then establish PLS-DA or OPLS-DA models after standardization and preprocessing, and finally evaluate reliability and identify key origin markers through VIP screening, permutation tests, and independent external validation.

### 4.2. The Performance of Classic Classifiers in Tea Traceability

After preprocessing and dimensionality reduction in the detection data, classification algorithms are needed to assign unknown samples to their respective origin categories. Linear Discriminant Analysis (LDA) finds the optimal projection direction by maximizing the ratio of between-class variance to within-class variance, and is suitable for scenarios where variables are approximately normally distributed and have similar covariance [65]. K-Nearest Neighbor (KNN) determines the category of a sample by majority voting, and has no strict assumptions about data distribution, making it particularly suitable for multi-class classification tasks [46,116,117]. Support Vector Machine (SVM) maps features to a high-dimensional space through kernel functions to maximize the margin between classes, and is adept at handling small sample sizes, high dimensions, and non-linear problems [117].

In recent years, several large-scale comparative studies have systematically evaluated the performance of three types of classifiers in the origin tracing of tea. Li et al. conducted a study on 791 black tea samples from 10 global production areas based on XRF elemental fingerprints, where the F1 score of SVM reached 87–97.7%, forming the first echelon of discrimination performance along with random forest and KNN [124]. In the task of narrow geographical scale origin tracing, which demands the highest resolution from classifiers, multiple classifiers have demonstrated remarkable classification accuracy. Liu et al., based on a multi-element database of 727 Taiwanese oolong tea samples, found that five types of classifiers, including LDA, Ridge regression, random forest, Boosting, and SVM, all achieved an accuracy rate of over 97% in distinguishing production areas [125].

LDA, KNN, and SVM are the most widely used classic classifiers in the traceability of tea origin. LDA is computationally efficient and the results are interpretable, making it suitable for elemental and isotopic data; KNN has no parameter assumptions and is suitable for volatile omics and spectral data, but it is sensitive to the K value and preprocessing; SVM is good at handling high-dimensional, small-sample, and nonlinear problems. A summary of representative applications of these classifiers in tea origin authentication is provided in Table 2. Each of the three types of classifiers has its own strengths and weaknesses. Direct comparisons of classifier performance between different studies should be treated with caution; differences in accuracy may be more due to variations in sample size, the range of production areas, and validation strategies rather than the inherent superiority or inferiority of the classifiers [83,126]. Therefore, under the current situation where methodological conditions are not standardized, the choice of classifier should be more based on the characteristics of the data rather than the absolute accuracy reported in the literature.

### 4.3. Advanced Applications of Machine Learning and Deep Learning

With the increase in data dimensions and the rise in multi-source integration, traditional classifiers struggle to handle complex challenges such as variable collinearity and nonlinear interactions. Ensemble learning and deep learning, with their advantages in automatic feature extraction, are emerging as new methodological engines for tea origin traceability.

Ensemble learning improves overall accuracy and robustness by combining multiple base learners. It mainly falls into two categories: one is the Bagging method represented by random forests, which builds decision trees in parallel through Bootstrap sampling and aggregates results by voting; the other is the Boosting method represented by XGBoost and LightGBM, which iteratively trains a sequence to correct errors. Bagging focuses on reducing variance to suppress overfitting, while Boosting focuses on hard-to-classify samples to improve accuracy. Both are good at handling high-dimensional nonlinear chemical data, especially suitable for the fusion modeling of multi-element and isotope fingerprints.

Random Forest is the most widely used ensemble learning method in the origin traceability of tea. In the study by Li et al. on 791 black tea samples from 10 geographical indication regions worldwide based on XRF elemental fingerprints, the F1 score of RF reached 87–97.7%, forming the first echelon of the best discrimination performance along with SVM, KNN, and MLP [124]. In the research by Guo et al. on the multi-spectral data fusion traceability of Rizhao green tea, RF also achieved a classification accuracy rate of 100% [53]. These studies collectively demonstrate that Random Forest exhibits stable high-performance across various data types, including elemental fingerprints and spectra. XGBoost and LightGBM, as representatives of the gradient boosting framework, are increasingly applied in the origin traceability of tea. XGBoost, with its regularization and parallel computing, achieved a qualitative accuracy rate of 93.3–100% in the identification of adulteration [127]. Although LightGBM started later, it has already shown superior classification performance to SVM, Random Forest, and XGBoost in electronic nose data [128]. Both are becoming important options in ensemble learning due to their efficient capture of nonlinear features. From the perspective of model selection strategies, Random Forest is robust to outliers and noise, insensitive to hyperparameters, and can output feature importance, making it suitable as a baseline algorithm for ensemble modeling; XGBoost and LightGBM have more prominent advantages in large sample and high-precision scenarios, but require fine-tuning to prevent overfitting.

Deep learning, with its core advantage of end-to-end automatic feature learning, overcomes the limitations of traditional machine learning that relies on manual feature engineering. In the context of tea origin traceability, the application of deep learning mainly unfolds along two paths: one is the classic neural networks represented by multi-layer perceptrons, which are suitable for nonlinear modeling of structured data; the other is the deep architectures represented by convolutional neural networks, which excel in spectral images, high-dimensional sequence data, and end-to-end feature extraction tasks. Zhao et al. systematically summarized the multi-dimensional applications of deep learning in tea variety, geographical origin, quality grade, fermentation stage, adulteration level, and chemical component monitoring, highlighting its end-to-end analysis capability as a powerful tool for precise tea quality detection [129].

The multi-layer perceptron (MLP) is the fundamental form of deep neural networks and has demonstrated outstanding classification capabilities on various types of data in the traceability of tea origins. In the study of Sri Lankan black tea origin, the feedforward backpropagation MLP achieved geographical authentication accuracy rates ranging from 46% to 82% for different production areas [130]. Peng et al. analyzed the volatile metabolites of 333 Wuyi rock tea samples using GC-TOF-MS, and the MLP model achieved an average accuracy of 92.7% on 176 features, with all independent test sets exceeding 90% [44]. These studies collectively indicate that the feature combination ability conferred by the multi-layer architecture of MLP is significantly superior to traditional linear models in handling complex nonlinear origin classification tasks.

Convolutional neural networks (CNNs) have significant advantages in the automatic feature extraction of spectral images. Chen et al. designed the ECA-ResNet, which integrates channel attention mechanisms, to achieve adaptive feature extraction of Raman and near-infrared spectra, achieving an accuracy rate of 95.05% in the multi-spectral fusion traceability of Pu’er tea [47]. Zhang et al. proposed a lightweight CNN model, Origin-Tea, which achieved an average accuracy rate of 92% on leaf images from 7 regions in Yunnan, with only 1.7 M parameters, reducing the parameter count by over 90% compared to CoAtNet, and an accuracy rate of 97% in independent village-level tests [131]. Liu et al. utilized hyperspectral imaging combined with CNN for the origin identification of Pu’er ripe tea, demonstrating that the deep learning performance based on the AlexNet model outperformed traditional machine learning methods [132].

In tea geographical origin authentication, it is essential to distinguish between linear and nonlinear classification approaches, as they differ fundamentally in assumptions, interpretability, data requirements, and performance on various analytical data types. Linear methods, such as linear discriminant analysis and partial least squares discriminant analysis, assume linear separability among geographical origin classes. They are computationally efficient, highly interpretable, and perform reliably with relatively small sample sizes, making them well-suited for structured data like elemental fingerprints and stable isotope ratios. However, they often fail to capture complex, non-linear interactions inherent in high-dimensional or collinear data. Nonlinear methods—including kernel-based support vector machines, k-nearest neighbors, random forest, and deep learning architectures—can model intricate non-linear relationships without strict distributional assumptions. They generally achieve higher accuracy when dealing with spectroscopic fingerprints, volatile profiles, or untargeted metabolomics data, but they typically require larger sample sizes to avoid overfitting and offer lower interpretability. The choice between linear and nonlinear models should therefore be guided by the characteristics of the analytical data, the available sample size, and the specific traceability task.

From the perspective of technical selection, MLP is suitable for structured data such as elemental fingerprints and biochemical components, while CNN is applicable to spatial or sequential data like hyperspectral images, spectral sequences, and leaf phenotype images. The performance of MLP tends to stabilize when the sample size reaches several hundred, while CNN can handle small and medium-sized samples with the aid of data augmentation. The “black box” nature of deep learning requires the use of tools such as attention visualization, feature map analysis, and SHAP to enhance interpretability in regulatory scenarios.

Although machine learning and deep learning have achieved high classification accuracy in the traceability of tea origin, they still generally face two major bottlenecks: overfitting with small samples and insufficient generalization ability across regions and years, which are particularly prominent in deep learning models with a large number of parameters. Zhao et al. pointed out that deep learning faces key challenges such as limited sample size, difficulty in fusing multi-source data, and lack of model interpretability in tea quality monitoring [129].

Overfitting in small sample sizes is the primary bottleneck restricting the large-scale application of deep learning in the traceability of tea origins. The sample sizes in most studies are only in the tens to hundreds, while the feature dimensions far exceed the number of samples, which easily leads to excellent performance of the model on the training set but a sharp decline in performance in independent external validation. For instance, Zheng et al. achieved a discrimination accuracy of 99% for the first and second grade production areas of West Lake Longjing, but with only tens of samples, and the cross-year robustness was not verified; Peng et al. achieved a 100% accuracy rate for township-level discrimination of Keemun black tea using FNN, but this was based on 39 differential metabolites and a limited sample size, with the risk of overfitting being significant [43,115]. To address the challenge of small sample sizes, strategies such as probabilistic machine learning and variational inference have been explored in other food traceability fields, and their modeling capabilities under small sample conditions provide methodological references for tea traceability [133,134].

The insufficient generalization ability of models across different tea-producing regions and years is another key bottleneck restricting the practical deployment of tea origin traceability. Most existing studies are based on modeling with samples from a single region and year, and the cross-scenario generalization performance lacks systematic evaluation. The strong fitting ability of deep learning is prone to capturing confounding factors irrelevant to the origin, leading to a sharp decline in performance when facing new batches of samples. Transfer learning is considered an effective path to break through geographical limitations. Lin et al. developed a transfer learning-based method in crop LIBS traceability, and the idea is of reference significance for tea traceability: after establishing a standardized database in major tea-producing regions, the models trained in data-rich regions can be quickly adapted to data-scarce regions, reducing the reliance on large-scale sampling for new regions [135].

**Figure 4:**
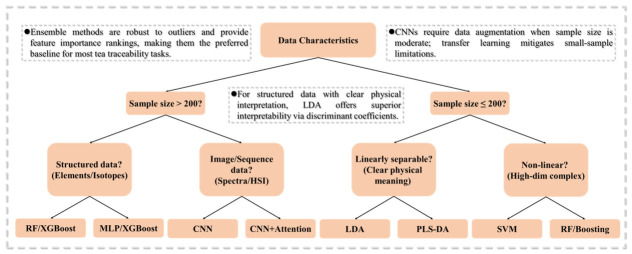
Decision tree for selecting appropriate machine learning models in tea geographical origin traceability based on data characteristics and task requirements.

Ensemble learning and deep learning represent the methodological frontier in the data analysis of tea origin traceability. Random forest has become the most mature ensemble tool in the fusion modeling of multi-element and isotopes; multi-layer perceptron demonstrates advantages in nonlinear modeling on structured data; convolutional neural networks possess end-to-end feature extraction capabilities in spectral images. Figure 4 presents the complete data analysis workflow, which incorporates a decision tree to guide model selection. Notably, overfitting in small sample sizes and insufficient generalization across scenarios constitute the core bottlenecks in the industrial application of deep learning. Most studies have sample sizes of only dozens to hundreds, with a prominent risk of overfitting under high-dimensional small sample conditions; the generalization performance across different tea-producing regions and years lacks systematic validation. Although strategies such as probabilistic machine learning and variational inference have been explored in other fields, they remain unexplored in tea. In the future, a standardized database covering major tea-producing regions and spanning multiple years should be established, and strategies such as transfer learning should be systematically introduced to verify their effectiveness in various tea types and regions.

### 4.4. Multi-Source Data Fusion Strategy

A single analytical technique can only capture chemical information in one dimension. However, the chemical composition of tea is influenced by multiple factors such as variety, origin, and processing, making it difficult for a single technique to comprehensively characterize the origin features. The data fusion strategy, through the collaborative analysis of multi-platform data, integrates complementary information and can effectively overcome the inherent limitations of a single technique, significantly improving the accuracy and robustness of the discrimination model.

The value-added benefits of multi-source data fusion in the traceability of tea origins have been verified by multiple studies. Li et al. achieved a 100% classification accuracy in the traceability of non-*Camellia sinensis* herbal teas by integrating multi-source chemical data with ensemble learning. The two-stage strategy they proposed, “feature selection first, then fusion decision-making,” holds methodological significance for tea tree leaves [136]. In the field of tea tree leaves, the decision-level fusion of FTIR-NIR-XRF for black tea, the spectral-level fusion of ^1^H NMR and NIR for green tea, and the joint modeling of isotopes and elements for oolong tea have all demonstrated that the fusion strategy can make up for the insufficiency of information dimensions in a single technology, achieving better classification accuracy and robustness in both narrow-scale and cross-regional tasks [42,109,137].

The core efficiency enhancement mechanism of data fusion lies in information complementarity, redundancy correction and confidence level improvement. However, this strategy still faces challenges such as insufficient data standardization, poor cross-platform comparability, “curse of dimensionality” and overfitting risks, as well as reduced model interpretability. Moreover, independent validation across laboratories is extremely scarce. In addition to improving accuracy, future fusion models should report uncertainty estimates and interpretable feature contributions. Tools such as SHAP, permutation importance, attention visualization, and stability selection can help determine whether the model relies on chemically meaningful geographical markers or on confounding variables such as cultivar, batch, storage, or processing intensity. In the future, a multi-source data standardization specification covering major tea-producing areas should be established, and small sample feature selection and fusion algorithms should be developed. These efforts, combined with the routine use of interpretability tools, will enhance both the credibility and regulatory acceptance of fusion models.

**Figure 5:**
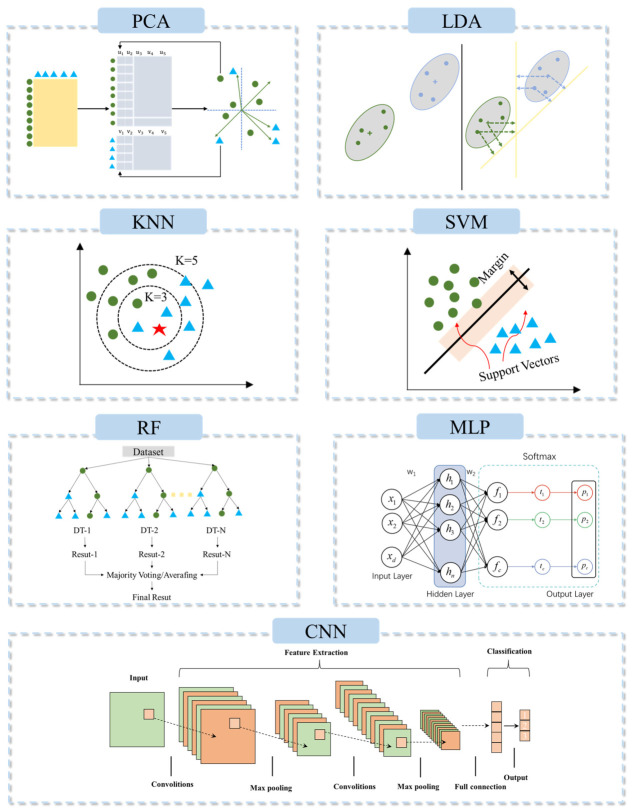
Schematic overview of machine learning and chemometric algorithms applied in tea geographical origin traceability, including unsupervised dimensionality reduction (PCA), classical classifiers (LDA, KNN, SVM), ensemble learning (RF), and deep learning architectures (MLP, CNN).

The various chemometric and machine learning algorithms described above, ranging from unsupervised dimensionality reduction (PCA) and classical classifiers (LDA, KNN, SVM) to ensemble learning (RF) and deep learning architectures (MLP, CNN), are schematically summarized in Figure 5. Together, these methods constitute the analytical backbone for transforming high-dimensional instrumental data into reliable geographical origin predictions. Beyond individual classifiers, ensemble learning and multi-source data fusion have demonstrated superior robustness in many tea origin studies. These advanced strategies help mitigate the limitations of single-technique or single-algorithm approaches, particularly when dealing with complex processing-induced chemical variations.

## 5. Conclusions

This review systematically combs through the entire technical chain of tea origin traceability, from detection principles, processing analysis, to data modeling. At the analytical technology level, stable isotopes and mineral element fingerprints, due to their robustness during processing, form the cornerstone of traceability. Spectroscopy and mass spectrometry-based metabolomics, with their high information density, are competent for micro-scale discrimination, while electronic noses/tongues and hyperspectral imaging offer portable supplements for high-throughput on-site screening. At the tea type specificity level, the differential masking of chemical fingerprints by the processing techniques of the six major tea types determines the applicable boundaries of the technology; green tea and white tea, with the least processing intervention, have the best traceability, while oolong tea and black tea, due to the fermentation process, undergo deep reshaping of their metabolic profiles, and post-fermentation of dark tea introduces multiple covariates such as fermentation degree, aging years, and tree age. Consistent with this, the degree of processing should be recognized as a primary covariate in tea-origin traceability: lightly processed teas are more amenable to metabolite-based discrimination, while heavily fermented or post-fermented teas demand more robust markers such as mineral elements, stable isotopes, or multi-source data fusion. At the data analysis level, from preprocessing and dimensionality reduction to classic classifiers, ensemble learning and deep learning, machine learning is driving tea origin traceability from empirical discrimination to intelligent decision-making. To ensure credibility, future studies should adopt a robust validation strategy including independent external and cross-year validation, adequate sample sizes, and transparent performance reporting. In summary, current research urgently needs to shift from “high-precision display” to “high-confidence verification”. Future research should move from proof-of-concept classification toward validated, transferable, and regulation-ready traceability systems. This requires multi-year and multi-region sampling, explicit control of cultivar and processing covariates, cross-laboratory validation, standardized spectral and metabolomic databases, interpretable machine-learning models, and uncertainty estimates rather than only reporting high classification accuracy.

## Figures and Tables

**Table 1:** Summary table of analytical techniques for tea origin traceability.

Technique Category	Representative Study	Key Platform/Elements	Tea Type	Main Finding	Reference
Stable isotope	Pilgrim et al.	δ^13^C, δ^15^N, trace elements	Multi-type	Processing has limited effect on isotope signals	[30]
	Liu et al.	δ^13^C, δ^15^N	Longjing green tea	C/N isotopes capture origin signals effectively	[31]
	Peng et al.	δ^13^C, δ^15^N	Keemun black tea	Variety/leaf age affects δ^15^N	[32]
	Xia et al.	δ^2^H, δ^18^O	Early-spring Longjing	Seasonal enrichment pattern of isotopes	[33]
	Li et al.	δ^13^C, δ^15^N, δ^2^H, δ^18^O	Pu-erh tea	Origin contribution dominant, but interaction with processing exists	[34]
Mineral element	Fernández-Cáceres et al.	multiple metals	Green/black tea	Lightly fermented teas less affected by processing	[35]
	Ma et al.	37 elements	Dongting Biluochun	LDA recognition rate 98.2%	[36]
	Zhao et al.	Na, Mg, Ca, Ni, Rb, Sr, Pb	Multi-type	Soil–tea element migration correlation	[37]
	Ren et al.	27 elements	Keemun black tea	LDA/SVM accuracy 100%	[38]
	Zhu et al.	Mo, Nd, Ce, Sr, Ba, V, Tm	Ripe Pu-erh	Pile-fermentation enriches 36 elements	[39]
	Zhu et al.	Mo, Cu, Rb	Anji Baicha	SVM prediction accuracy 92.7%	[40]
Spectral/MS metabolomics	Yan et al.	NIR, PLS-DA	Anxi Tieguanyin	First rapid authentication feasibility	[41]
	Meng et al.	^1^H NMR, NIR	Tieguanyin	Fusion accuracy 86.2–95.8%	[42]
	Zhang et al.	UHPLC-QTOF-MS	West Lake Longjing	Monte Carlo 99% prediction accuracy	[43]
	Peng et al.	GC-TOF-MS	Wuyi rock tea	MLP average accuracy 92.7%	[44]
	Hou et al.	^1^H NMR, RF	Longjing tea	RF accuracy 92.2%, LDA 85.6%	[45]
	Li et al.	FTIR/NIR, SVM/KNN	Black tea (9 origins)	100% cross-validation accuracy	[46]
	Chen et al.	Raman, NIR, ECA-ResNet	Pu-erh tea	Multi-spectral fusion accuracy 95.05%	[47]
Emerging sensing	Yan et al.	Electronic tongue	Anji Baicha	PLS-DA outperforms PCA	[48]
	Hong et al.	HSI	Longjing tea	>84% accuracy, origin prediction map	[49]
	Liu et al.	NIR-HIS, PCA-SVM	Green tea (3 origins)	Origin accuracy 97.5%, month 95%	[50]
	Kanaga Raj et al.	Impedimetric e-tongue, PLS-DA/PLSR	Black tea	Multi-sensor electronic tongue	[51]
	Jin et al.	E-nose, GC-MS	Tongcheng Xiaohua tea	7 regional differential volatiles	[52]
	Guo et al.	NIR, HIS, SVM/RF	Rizhao green tea	Fusion accuracy 100%	[53]

**Table 2:** Summary of classic classifiers for tea origin authentication.

Classifier	Representative Studies	Tea Type	Key Performance	References
LDA	Ma et al.	Dongting Biluochun	Recognition rate 98.2%	[36]
	Liu et al.	Multiple tea types	Effective origin discrimination	[65]
	Kaushal et al.	Oolong tea	Overall accuracy 98.33%	[108]
KNN	Li et al.	Black tea (9 origins)	Cross-validation accuracy 100%	[46]
	Zhang et al.	White tea	Accuracy 88.97–97.96%	[100]
	Yun et al.	Black tea (China, India, Sri Lanka)	Discrimination rate 95–100%	[116]
SVM	Li et al.	Black tea (9 origins)	Cross-validation accuracy 100%	[46]
	Lou et al.	Wuyi rock tea	Accuracy 97.73%	[109]
	Zhu et al.	Anji Baicha	Independent test prediction accuracy 92.7%	[40]

## Data Availability

No new data were created or analyzed in this study. Data sharing is not applicable to this article.
